# Aminotransferase-to-lymphocyte ratio as a valuable prognostic marker for patients with stage I-III colorectal cancer: a retrospective study

**DOI:** 10.3389/fonc.2024.1446557

**Published:** 2024-11-18

**Authors:** Hailun Xie, Lishuang Wei, Shuangyi Tang, Jialiang Gan

**Affiliations:** ^1^ Department of Gastrointestinal and Gland Surgery, The First Affiliated Hospital, Guangxi Medical University, Nanning, China; ^2^ Guangxi Key Laboratory of Enhanced Recovery after Surgery for Gastrointestinal Cancer, The First Affiliated Hospital, Guangxi Medical University, Nanning, China; ^3^ Department of Pharmacy, The First Affiliated Hospital, Guangxi Medical University, Nanning, China; ^4^ Department of Colorectal and Anal Surgery, The First Affiliated Hospital, Guangxi Medical University, Nanning, China

**Keywords:** prognostic nomograms, aminotransferase-to-lymphocyte ratio, colorectal cancer, survival, sarcopenia, postoperative complications

## Abstract

**Background:**

There are no population-based studies on the prognostic value of the preoperative aminotransferase-to-lymphocyte ratio (AALR) in predicting recurrence and survival in patients with colorectal cancer (CRC) who have undergone curative resection.

**Aim:**

This study explored the relationship between AALR and prognosis of CRC patients, specifically stage III CRC.

**Methods:**

Restricted Cubic Splines were used to evaluate the relationship between AALR and outcomes. The survival curve was generated using the Kaplan-Meier method and the log-rank test. COX regression analysis was used to identify the independent prognostic factors of CRC patients. Logistic regression analysis was used to assess the independent risk factors affecting sarcopenia and postoperative complications. Concordance index and calibration curves were used to evaluate the discriminative ability of the prognostic nomograms. Finally, according to a ratio of 7:3, the total population was randomized into two cohorts to validate the practicability of the prognostic nomograms.

**Results:**

In total, 1304 stage I-III CRC were enrolled in this study. There was a significant positive correlation between AALR and PFS/OS in CRC patients. The PFS/OS ratio of the high AALR group was significantly lower than that of the low AALR group. In the subgroup analysis, we found that the AALR significantly stratified the prognosis of patients with stage III CRC. A high AALR was still independently associated with poor PFS (HR = 1.335, 95% CI =1.075–1.657, p=0.009) and OS (HR = 1.382, 95% CI =1.139–1.677, p=0.001) in CRC patients. Variables with a value ≤ 0.05 in multivariable analysis were incorporated into the construction of prognostic nomograms for predicting 1-5 years PFS/OS of CRC patients. The results of the concordance index and calibration curves confirmed that these prognostic nomograms had a good prediction accuracy. In addition, we demonstrated the good predictive performance of these nomograms in a randomized internal validation cohort.

**Conclusion:**

AALR is an effective prognostic marker for predicting long-term outcomes and could provide a valuable reference for sarcopenia and postoperative complications in CRC patients. AALR-based nomograms have good predictive accuracy and can help to develop individualized risk stratification, follow-up, and treatment strategies for CRC patients.

## Introduction

The overall incidence of global colorectal cancer (CRC) has risen to third, and its overall mortality has risen to second for global cancer death (1). In China, the population’s burden of CRC is also not optimistic, with the incidence of CRC jumping to second and mortality to fifth, and these numbers continue to rise (2). If the prognosis of CRC patients can be accurately predicted, personalized postoperative treatment and follow-up plans can be developed, which can reduce both the financial burden and the side effects of drugs, improve the quality of life, and extend the survival time of patients. Despite advances in diagnostic and surgical techniques, clinical prognosis of patients with advanced CRC remains poor. The five-year survival rate of patients with early CRC can reach 90% after surgery, while five-year survival rate of patients with advanced CRC is less than 20% (3, 4). The American Joint Committee on Cancer (AJCC) staging system, including treatment recommendations, has been widely validated and is the most commonly used staging method for CRC patients (5, 6). Owing to the heterogeneity of tumors, staging systems have certain limitations. Even with the same staging, the patient’s prognosis may differ. Serum carcinoembryonic antigen (CEA), a common tumor biomarker, is also considered an effective indicator for predicting the prognosis of CRC patients. However, more than 50% of CRC patients remain within the normal range (7, 8). Therefore, more effective prognostic indicators need to be explored to facilitate active intervention and increase the survival rate.

As an important characteristic of metastatic disease, inflammation promotes their occurrence and development (9, 10). Many hematological components have been combined to form inflammation-based prognostic scores to predict the prognosis of CRC patients (11–13). Recently, the aminotransferase-to-lymphocyte ratio (AALR) has attracted increased attention. Previous studies have reported that the AALR is a promising biomarker for evaluating the prognosis of hepatocellular carcinoma (HCC). A meta-analysis by Peng et al. (14), showed that an elevated AALR in patients with HCC predicted lower survival outcomes and was strongly associated with some important features of HCC. Qiu et al. (15), found that AALR combined with a CA199 blood test could effectively stratify the prognosis of intrahepatic cholangiocarcinoma. Chen et al. (16) also found that preoperative AALR was a non-invasive, simple, and effective indicator for the prognostic evaluation of HCC patients.

CRC patients often experience reduced food intake, poor nutrient absorption, and systemic inflammatory responses. This leads to a state of high catabolism and low anabolism, resulting in progressive weight loss, including decreased muscle mass, reduced muscle strength, and impaired muscle function. Consequently, this can lead to sarcopenia, which is associated with poorer prognosis. Sarcopenia significantly affects the postoperative quality of life and prognosis of patients, potentially leading to prolonged hospital stays, increased medical costs, and even higher mortality rates. It is a well-known independent risk factor for poor outcomes in CRC patients (17–20). Inflammation is closely linked to sarcopenia, with chronic inflammation considered a key mechanism in its development (21–23). Therefore, AALR may also serve as a promising biomarker for predicting sarcopenia in CRC patients.

However, to our knowledge, there are no population-based studies on the prognostic value of preoperative AALR for predicting sarcopenia, recurrence, and survival in CRC patients who have undergone curative resection. The liver is well known to be the most common site of metastasis in CRC. Cancer cells often migrate to the liver through blood from the portal vein. Therefore, abnormal liver function in CRC patients may reflect hidden metastasis and poor prognosis. AALR combines aminotransferase and lymphocyte levels, which reflect liver function and inflammatory immune status, respectively, and may be a promising prognostic biomarker for CRC patients. This study aimed to explore the relationship between AALR and the prognosis of CRC patients, specifically stage III CRC. In addition, we developed a novel AARI-based prognostic model to accurately predict 1-5 years of clinical outcomes in CRC patients, providing a reference for individualized monitoring of CRC patients after surgery.

## Patients and methods

### Patients

In an observational study, from January 2012 to December 2015, patients with stage I-III CRC who received surgical treatment were retrospectively recruited from the Department of Colorectal and Anal Surgery, First Affiliated Hospital of Guangxi Medical University. Patients met the following inclusion criteria: 1) pathologically confirmed diagnosis of CRC, 2) primary tumors, 3) curative resection with a negative surgical margin, and 4) complete clinicopathological characteristics. Patients with hepatitis, liver disease, or obvious abnormal liver function; other malignancies, AIDS, recent acute infection, or high fever; or received neoadjuvant chemoradiotherapy were excluded.

### Data collection

Routine examination within one week before surgery: routine blood test, blood biochemical series test, chest X-ray examination, abdominal ultrasound examination, computer tomography (CT), or magnetic resonance imaging (MRI). Clinicopathological characteristics included demographic data, medical history, whole blood count, aspartate aminotransferase (AST) and serum CEA levels, tumor-nodes-metastasis stage (TNM stage), pathological tumor stage (pT stage), pathological node stage (pN stage), perineural invasion, vascular invasion, macroscopic type, differentiation, tumor location, tumor size, radiotherapy, chemotherapy, length of stay, and hospitalization cost. The TNM stage was based on AJCC cancer staging 8th edition. Body mass index (BMI) was defined as the weight (kg)/height squared (m^2^). Normal CEA, <5.00 ng/ml; high CEA, ≥5.00 ng/ml. The equation for the AALR is as follows: AST/lymphocyte ratio.

### Follow-up

The follow-up assessment included physical and blood tests, including tumor markers, and imaging examination (chest, abdomen, and pelvic CT or MRI. This was performed every 3 months in the first two years and then once every 6 months until 5 years after surgery. Colonic endoscopy was performed annually. The most recent follow-up date was February 2022. The median follow-up time was 67.37 months (95% CI:55.88 months -79.86 months).

### Outcome

The main outcomes of this study were progression-free survival (PFS) and overall survival (OS). Secondary outcomes were preoperative sarcopenia and postoperative complications. PFS refers to the time between the date of surgery and the patient’s disease recurrence, death, or the last follow-up. OS was defined as the time between the date of surgery and the patient’s death from any cause. According to the 2019 Asian Working Group on Sarcopenia (AWGS) diagnostic consensus on sarcopenia (24), skeletal muscle index (SMI) < 6.92 kg/m^2^ in a man, and SMI < 5.13 kg/m^2^ in a woman is considered sarcopenia. SMI was assessed using the validated equation of the Chinese population (25): SMI = 0.193 ×  body weight (kg) + 0.107 × height (cm) − 4.157 × sex (male = 1, female = 2)  − 0.037 × age (years) − 2.631. Postoperative complications in CRC patients were strictly classified according to the modified Clavien complication grading system (26).

### Statistical analysis

Continuous variables were summarized using median (interquartile difference [IQRs]) or mean (standard deviation [SD]). The classification variable was summarized as frequency (percentage). The t-test was used to test the associations between continuous variables. The chi-square test or Fisher’s exact test was used to test the associations between categorical variables. Based on the survival status of CRC patients, the optimal stratification method was used to determine the optimal threshold value of AALR. Restricted Cubic Splines (RCS) were used to evaluate the relationship between AALR and the outcomes of CRC patients. The survival curve was generated using the Kaplan-Meier method and the log-rank test. Univariate and multivariable analyses were performed to identify independent prognostic factors using the COX regression analysis. Logistic regression analysis was used to assess independent risk factors affecting sarcopenia and postoperative complications in CRC patients. Variables with a value ≤0.05 in multivariable analysis were incorporated into the construction of prognostic nomograms for predicting 1-5 years PFS/OS of CRC patients. The concordance index (C-index) was used to evaluate the discriminative ability of nomograms. Calibration curves were constructed to compare the predicted probability of PFS/OS with actual results. The model was verified internally through 1000 resampling. Finally, according to a ratio of 7:3, the total population was randomized into two cohorts to validate the practicability of the prognostic nomograms. In this study, a two-tailed p value less than 0.05 was considered statistically significant. Statistical analysis was performed using the R language version 4.0.2 (http://www.R-project.org).

## Results

### Demographic and clinicopathological characteristics

The baseline clinicopathological characteristics of the patients were shown in **Table 1**. Among the 1304 enrolled patients, 821 (63.0%) were male and 483 (37.0%) were female. There were 284 cases (21.8%) in stage I, 480 (36.8%) in stage II, and 540 (41.4%) in stage III. The median age at CRC diagnosis was 58.31 years (± SD:13.00). There were 687 patients (52.7%) with rectal cancer and 617 (47.3%) with colon cancer. The median maximum tumour size was 4.50 cm (range 3.50 to 6.00 cm). Serum CEA levels were elevated in 494 CRC patients (37.9%). A total of 122 patients (9.4%) had perineural invasion, and 208 patients (16.0%) had vascular invasion. According to the optimal stratification method, the optimal AALR threshold for predicting the postoperative prognosis of CRC patients was 16.43 (**Supplementary Figure S1**). All patients were then divided into two groups: a high AALR group (≥16.43, n=234) and a low AALR group (<16.43, n=1070). During the follow-up period, a total of 457 patients died, of whom 278 (60.8%) succumbed to disease recurrence and metastasis, while the remaining 179 (39.2%) died from other causes. Patients with high AALR had a higher risk of mortality compared to those with low AALR (41.5% vs 33.6%, p=0.028).

**Table 1:** The relationships between the AALR and clinicopathological characteristics of CRC patients.

Clinicopathological characteristics	Overall (n = 1304)	AALR		P value
		Low (n = 1070)	High (n = 234)	
Sex(Man)	821 (63.0)	657 (61.4)	164 (70.1)	0.016
Age (mean (SD))	58.31 (13.00)	58.07 (12.83)	59.42 (13.71)	0.150
BMI (median [IQR])	22.07 (20.00, 24.45)	22.15 (20.08, 24.57)	21.78 (19.53, 23.95)	0.017
Hypertension (Yes)	218 (16.7)	178 (16.6)	40 (17.1)	0.941
Diabetes (Yes)	82 (6.3)	72 (6.7)	10 (4.3)	0.210
T stage (T3-4)	940 (72.1)	767 (71.7)	173 (73.9)	0.539
N stage				0.890
N0	764 (58.6)	627 (58.6)	137 (58.5)	
N1	351 (26.9)	290 (27.1)	61 (26.1)	
N2	189 (14.5)	153 (14.3)	36 (15.4)	
TNM stage (III-IV)				0.849
Stage I	284 (21.8)	236 (22.1)	48 (20.5)	
Stage II	480 (36.8)	391 (36.5)	89 (38.0)	
Stage III	540 (41.4)	443 (41.4)	97 (41.5)	
Perineural invasion (Yes)	122 (9.4)	102 (9.5)	20 (8.5)	0.730
Vascular invasion (Yes)	208 (16.0)	170 (15.9)	38 (16.2)	0.973
Macroscopic type				0.593
Protrude type	375 (28.8)	313 (29.3)	62 (26.5)	
Infiltrating type	102 (7.8)	81 (7.6)	21 (9.0)	
Ulcerative type	827 (63.4)	676 (63.2)	151 (64.5)	
Differentiation (Poor)	168 (12.9)	129 (12.1)	39 (16.7)	0.072
Tumor location (Rectal)	687 (52.7)	565 (52.8)	122 (52.1)	0.910
Tumor size (median [IQR])	4.50 (3.50, 6.00)	4.50 (3.50, 6.00)	4.00 (3.00, 5.88)	0.150
CEA (High)	494 (37.9)	396 (37.0)	98 (41.9)	0.188
Radiotherapy (Yes)	124 (9.5)	83 (7.8)	41 (17.5)	<0.001
Chemotherapy (Yes)	581 (44.6)	474 (44.3)	107 (45.7)	0.745
Death (Yes)	457 (35.0)	360 (33.6)	97 (41.5)	0.028
Length of stay (median [IQR])	17.00 (11.00, 21.00)	16.50 (11.00, 21.00)	17.00 (11.00, 21.00)	0.346
Hospitalization cost (median [IQR])	49360.30 (44621.77, 55679.28)	49490.16 (44678.61, 55603.47)	48897.21 (44488.32, 56621.56)	0.673

CRC, colorectal cancer; BMI, body mass index; AALR, aminotransferase-to-lymphocyte ratio.

### Relationship between AALR and PFS in CRC patients

**Figure 1:**
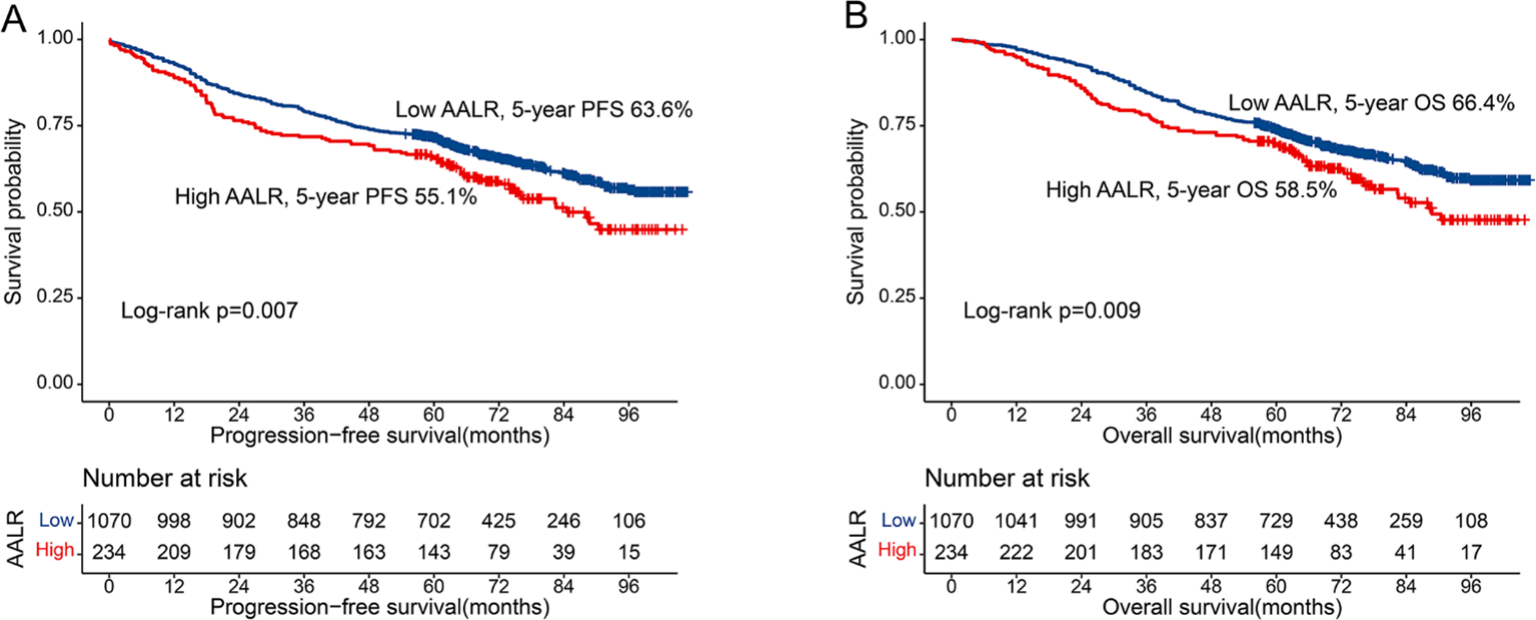
Kaplan-Meier curve of AALR in CRC patients. **(A)** Progression-free survival; **(B)** Overall survival.

**Figure 2:**
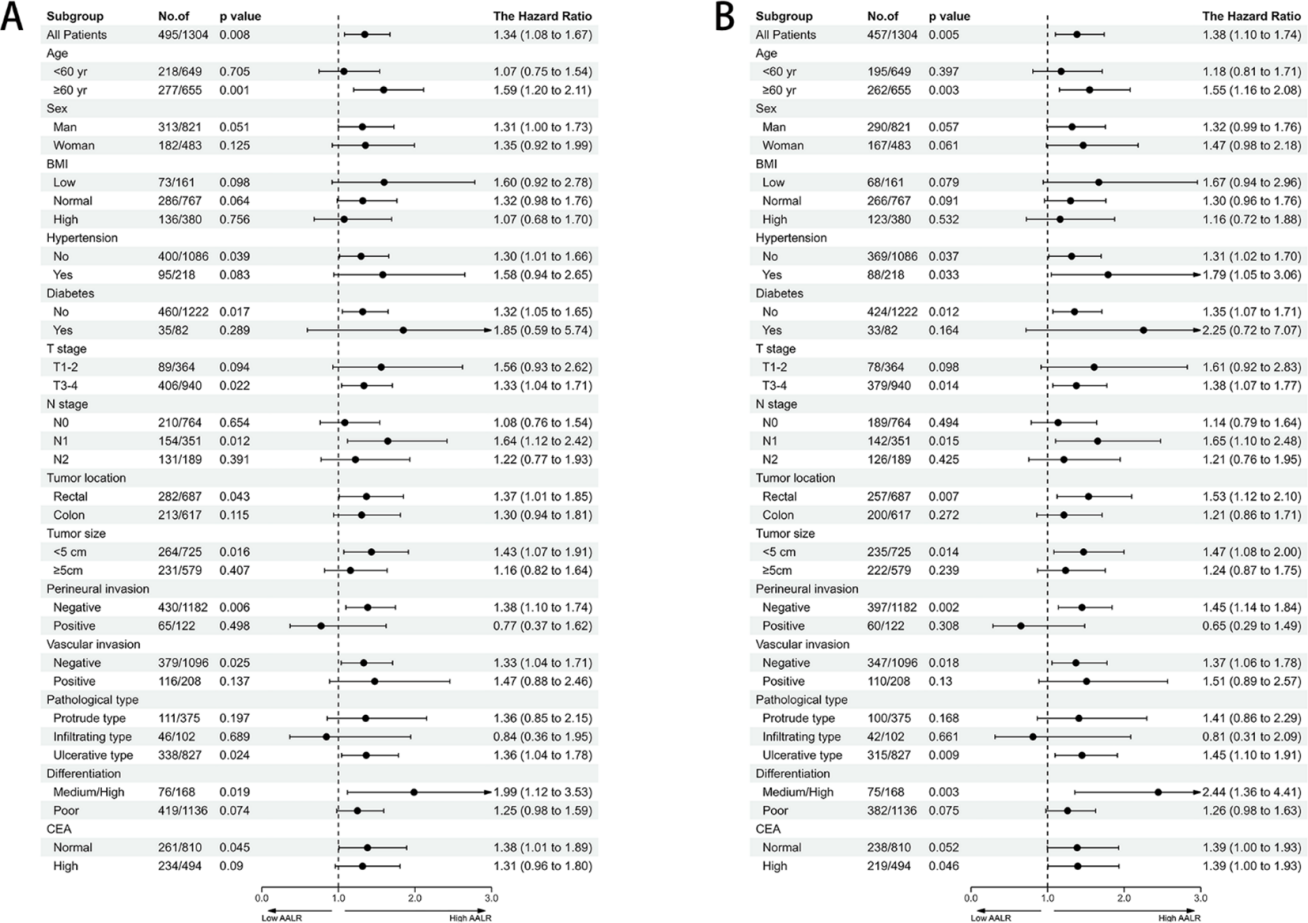
The association between AALR and hazard risk of survival in various subgroups. **(A)** Progression-free survival, **(B)** Overall survival). adjusted for gender, age, BMI, hypertension, diabetes, T stage, N stage, tumor location, tumor size, perineural invasion, vascular invasion, macroscopic type, differentiation, radiotherapy, chemotherapy.

After adjusting for confounding factors, there was a positive linear relationship between the AALR and adverse PFS in CRC patients (**Supplementary Figure S2A**). During follow-up, 308 patients (23.6%) developed recurrence, including 245 patients in the low AALR group (22.9% of the total low AALR group) and 63 patients in the high AALR group (26.9% of the total high AALR group). The Kaplan-Meier survival curve showed that PFS in the high AALR group was significantly lower than that in the low AALR group (55.1% vs. 63.6%, p=0.007) (**Figure 1A**). In the subgroup analysis, we found that AALR significantly stratified the prognosis of patients with stage III CRC (35.1% vs. 49.7%, p=0.008). However, no significant difference was observed in patients with stage I-II CRC (**Supplementary Figures S3A-C**). Univariate COX regression analysis showed that CRC patients with a high AALR had a 1.344 times greater risk of adverse PFS than CRC patients with a low AALR (HR = 1.344, 95% CI =1.084–1.668, p=0.007). In the subsequent multivariable COX regression analysis, high AALR was still independently associated with poor PFS in CRC patients (HR = 1.335, 95% CI =1.075–1.657, p=0.009) (**Table 2**). Fourteen clinical features (31 subgroups) were included in the multivariable Cox regression analysis. We found that a high AALR was a risk factor for PFS in CRC patients in most subgroups (**Figure 2A**).

**Table 2:** Univariate and multivariate Cox regression analysis of clinicopathological characteristics associated with progression-free survival in CRC patients.

Clinicopathological characteristics	Progression-free survival			
	Univariate analysis		Multivariate analysis	
	HR (95%CI)	P value	HR (95%CI)	P value
Age	1.356 (1.135-1.619)	0.001	1.496 (1.246 - 1.797)	<0.001
T stage (T3-4)	2.034 (1.617-2.558)	<0.001	1.491 (1.167 - 1.905)	0.001
N stage		<0.001		<0.001
N0	Ref.			
N1	1.798 (1.46-2.214)	<0.001	1.607 (1.296 - 1.992)	<0.001
N2	4.048 (3.25-5.042)	<0.001	3.264 (2.57 - 4.145)	<0.001
Perineural invasion (Positive)	1.678 (1.292-2.179)	<0.001	1.006 (0.752 - 1.345)	0.970
Vascular invasion (Positive)	1.995 (1.619-2.457)	<0.001	1.433 (1.129 - 1.818)	0.003
Pathological type		0.006		0.119
Protrude type				
Infiltrating type	1.539 (1.091-2.17)	0.014	1.439 (1.018 - 2.033)	0.039
Ulcerative type	1.387 (1.119-1.719)	0.003	1.131 (0.907 - 1.41)	0.273
Differentiation (High-medium)	0.699 (0.548-0.893)	0.004	0.849 (0.66 - 1.092)	0.201
Tumor location (Colon cancer)	0.800 (0.67-0.956)	0.014	0.777 (0.648 - 0.932)	0.007
CEA (≥5ng/ml)	1.7 (1.425-2.029)	<0.001	1.434 (1.195 - 1.722)	<0.001
AALR (High)	1.344 (1.084-1.668)	0.007	1.335 (1.075 - 1.657)	0.009

CRC, colorectal cancer; BMI, body mass index; AALR, aminotransferase-to-lymphocyte ratio.

### Relationship between AALR and OS in CRC patients

There was a significant positive correlation between AALR and OS in CRC patients; that is, with an increase in AALR, the risk of adverse prognosis in CRC patients gradually increased (**Supplementary Figure S2B**). During follow-up, 457 patients (23.6%) died, including 360 in the low AALR group (33.6% of the total low AALR group) and 97 in the high AALR group (41.5% of the total high AALR group). The OS of the high AALR group was significantly lower than that of the low AALR group (58.5% vs. 66.4%, P =0.009) (**Figure 1B**). In addition, we performed a subgroup survival analysis of OS based on TNM stage. For stage I-II CRC patients, although patients in the high AALR group had a lower prognosis than those in the low AALR group, no significant difference was observed (**Supplementary Figures S3D, E**). For stage III CRC patients, the OS in the high AALR group was significantly lower than that in the low AALR group (40.2% vs. 52.6%, p<0.001) (**Supplementary Figure S3F**). In univariate COX regression analysis, AALR was closely correlated with the OS of CRC patients (HR = 1.723, 95% CI =1.434–2.071, p<0.001). Multivariable COX regression analysis suggested that AALR was independently associated with the OS of CRC patients (HR = 1.382, 95%CI, 1.139–1.677; p=0.001) (**Table 3**). In the subgroup multivariate Cox regression analysis of OS, a high AALR was a risk factor for OS in most of the subgroups (**Figure 2B**).

**Table 3:** Univariate and multivariate Cox regression analysis of clinicopathological characteristics associated with overall survival in CRC patients.

Clinicopathological characteristics	Overall survival			
	Univariate analysis		Multivariate analysis	
	HR (95%CI)	P value	HR (95%CI)	P value
Age	1.453 (1.206-1.749)	<0.001	1.598 (1.319 - 1.935)	<0.001
T stage (T3-4)	2.129 (1.668-2.717)	<0.001	1.469 (1.133 - 1.905)	0.004
N stage		<0.001		<0.001
N0	Ref.			
N1	1.805 (1.452-2.244)	<0.001	1.604 (1.281 - 2.009)	<0.001
N2	4.144 (3.303-5.199)	<0.001	3.425 (2.679 - 4.378)	<0.001
Perineural invasion (Positive)	1.633 (1.244-2.143)	<0.001	0.965 (0.712 - 1.308)	0.820
Vascular invasion (Positive)	2.024 (1.632-2.509)	<0.001	1.419 (1.11 - 1.815)	0.005
Pathological type		0.007		0.123
Protrude type				
Infiltrating type	1.633 (1.244-2.143)	<0.001	1.457 (1.015 - 2.094)	0.042
Ulcerative type	2.024 (1.632-2.509)	<0.001	1.153 (0.915 - 1.453)	0.227
Differentiation (High-medium)	0.643 (0.502-0.824)	<0.001	0.787 (0.609 - 1.016)	0.066
CEA (≥5ng/ml)	1.218 (1.014-1.464)	0.035	1.1 (0.91 - 1.329)	0.327
AALR (High)	1.723 (1.434-2.071)	<0.001	1.382 (1.139 - 1.677)	0.001

CRC, colorectal cancer; BMI, body mass index; AALR, aminotransferase-to-lymphocyte ratio.

### Relationship between AALR and sarcopenia in CRC patients

In this study, 241 CRC patients (18.5%) were diagnosed with sarcopenia. The incidence of sarcopenia in CRC patients with a low AALR was 16.9% compared to 25.6% in CRC patients with a high AALR. Univariate logistic regression analysis showed that a high AALR was significantly associated with sarcopenia in CRC patients (OR = 1.694, 95%CI, 1.213–2.366; p=0.002). Multivariable logistic regression analysis demonstrated that a high AALR was an independent risk factor for predicting sarcopenia in CRC patients (OR = 1.765, 95% CI =1.120–2.781, p=0.002) (**Supplementary Table S1**).

### Relationship between AALR and postoperative complications in CRC patients

A total of 267 CRC patients (20.5%) experienced some degree of postoperative complications. There were 132 cases of grade I complications, 98 of grade II complications, 16 of grade IIIa complications, 10 of grade IIIb complications, 6 of grade IVa complications, and 4 of grade IVb complications. The incidence of postoperative complications in CRC patients in the low AALR group was 18.6%, while the incidence of postoperative complications in CRC patients in the high AALR group was 29.1%. Compared with patients with a low AALR, CRC patients in the high AALR group had an approximately 1.7 times higher risk of postoperative complications (OR = 1.793, 95% CI =1.300–2.473, p<0.001). Multivariable logistic regression analysis indicated that a high AALR was an independent risk factor for postoperative complications in CRC patients (OR = 1.781, 95% CI =1.286–2.468, p=0.001) (**Supplementary Table S2**).

### Construction of the prognostic prediction models

**Figure 3:**
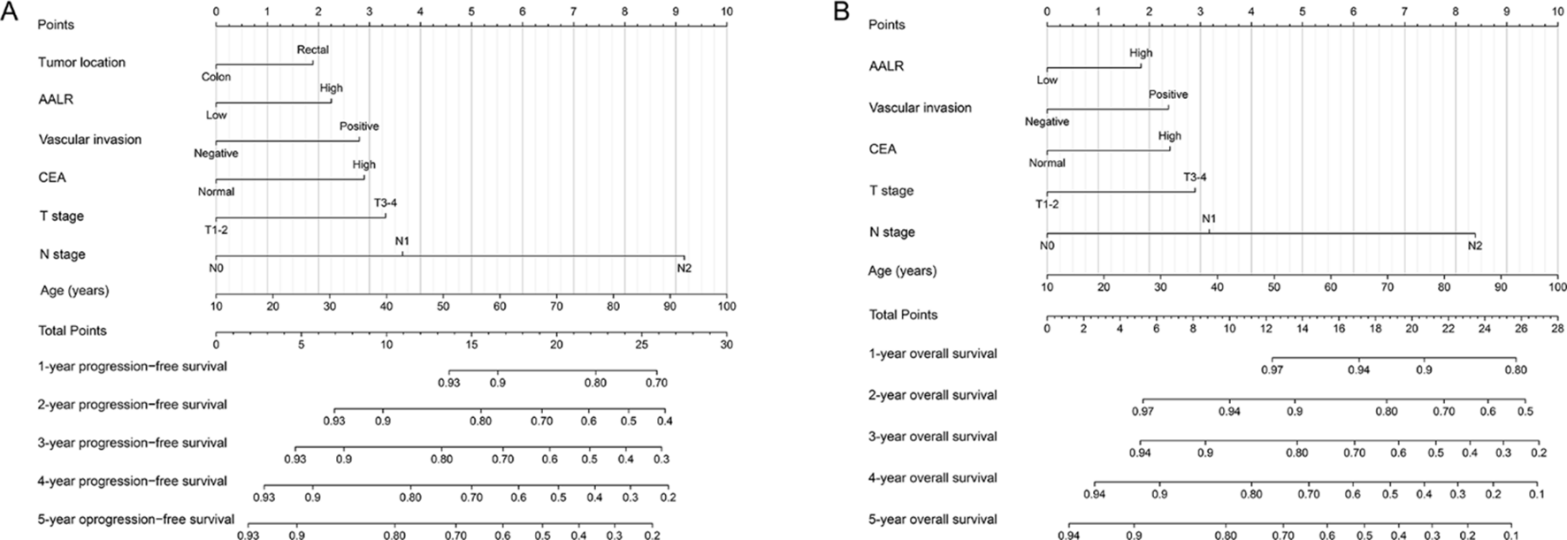
Construction prognostic nomograms in CRC patients. **(A)** The progression-free survival nomogram; **(B)** The overall survival nomogram.

We constructed a PFS nomogram (including age, T stage, N stage, vascular invasion, tumor location, CEA level, and AALR) based on all independent indicators in multivariable COX regression analysis of PFS (**Figure 3A**). The higher the total score, the worse the clinical prognosis. This nomogram can be used to predict 1-5 years PFS of CRC patients after surgery. The C-index of this nomogram was 0.686 (95% confidence interval [CI]:0.674–0.698). The three - and 5-year calibration curves showed good agreement between the predicted and observed values (**Supplementary Figures S4A, B**). Similarly, we included significant variables in the multivariable COX regression analysis of OS to construct an OS nomogram, including age, T and N stages, vascular invasion, CEA level, and AALR (**Figure 3B**). The C-index of the OS nomogram was 0.689 (95% CI:0.665-0.714). The calibration diagram demonstrated the best agreement between the predicted survival probability and actual observed values (**Supplementary Figures S4C, D**). The DCA showed that AALR-based nomograms provided better clinical benefits than traditional TNM stage for both PFS and OS in the 1–5 year period (**Supplementary Figures S5A, B**). We then performed a randomized internal validation. All patients were randomly assigned to two cohorts: validation cohort A (916 cases) and validation cohort B (388 cases) (using computer-generated random numbers). There was no significant difference in the clinicopathological characteristics between validation cohorts A and B (**Supplementary Table S3**). In validation cohort A, the C-indices of PFS and OS nomograms were 0.682 (95%CI:0.654-0.710) and 0.685 (95%CI:0.655-0.715), respectively. In validation cohort B, the C-indices of PFS and OS nomograms were 0.715 (95%CI:0.675-0.755) and 0.723 (95%CI:0.680-0.766), respectively. In addition, the calibration plots of PFS (**Supplementary Figure S6A**) and OS (**Supplementary Figure S6B**) at 3 and 5 years after surgery showed good agreement between the predicted and observed values in the validation datasets.

## Discussion

In the present study, we demonstrated for the first time that preoperative AALR is a potential predictor of PFS and OS in CRC patients, particularly in patients with stage III CRC. We also found that high AALR was an independent risk factor for predicting sarcopenia and postoperative complications in CRC patients. We constructed AALR-based prognostic nomograms to predict 1-5year PFS/OS in CRC patients. In addition, we demonstrated the good predictive performance of these nomograms in an internal validation cohort. These nomograms can provide individualized recommendations for the prognosis assessment of CRC patients.

Liver function plays an important role in the development of CRC. Most CRC deaths are associated with distant spread to the liver (27). Several large-scale studies have associated liver function markers with an increased risk of developing CRC (28). AST is a commonly used biomarker for hepatocyte injury (29). AST is also an important component of glycolysis in tumor cells, and abnormal tumor metabolism may lead to an increase in AST (30, 31). Tumor progression is associated with increased proliferation, cell turnover, tissue damage, and necrosis. These pathological processes might lead to elevated AST levels. In addition, during tumorigenesis and development, tumor cells can trigger intracellular inflammatory processes, and systemic chronic inflammation in turn can promote tumor angiogenesis and inhibit tumor apoptosis (32, 33). In addition, systemic chronic inflammation can induce tumor epithelial-mesenchymal transition (EMT) or reactive oxygen species (ROS), which are chemical substances that eventually lead to tumor formation, proliferation, metastasis and recurrence (34). Wandering and infiltrating lymphocytes reflect the patient’s inflammatory state. In addition, lymphocytes are an important cellular component of the immune response and are involved in antitumor immune processes. The increase in tumor lymphocytes is related to the improvement of patient prognosis (35, 36). Combining the advantages of both, the AALR may be a promising prognostic marker in CRC patients.

Currently, clinicians still rely on routine pathological diagnoses such as tumor size, TNM stage, and distant metastasis. However, these depend on either expensive whole-body imaging or invasive surgical procedures. Therefore, it is of great significance to identify non-invasive biochemical markers for CRC patients. In this study, we found that the AALR was a useful predictor of long-term outcomes in CRC patients and had a good stratification effect in most subgroups. We found that patients with high AALR had poor DFS and OS, especially stage III patients, suggesting that AALR may be more suitable for patients with advanced CRC and minor metastases. These results indicate that the AALR is a reliable, objective, and effective prognostic indicator for CRC patients and can serve as a useful prognostic reference for CRC patients.

High systemic inflammation increases the risk of malnutrition and postoperative complications in CRC patients. In our study, sarcopenia was present in approximately 16.9% of the CRC patients. A high AALR was an independent risk factor for sarcopenia in CRC patients. An increase in systemic inflammation could lead to the intensification of malnutrition in patients, leading to sarcopenia and even cachexia (37, 38). Postoperative complications could delay the length of hospital stay, increase the cost of hospitalization, reduce quality of life, and negatively affect patient survival of patients (39, 40). In this study, approximately 20.5% of the CRC patients experienced postoperative complications. Multivariable logistic regression analysis showed that a high AALR was independently associated with postoperative complications in CRC patients.

Single clinical indicators have certain limitations and may not comprehensively reflect the patient’s prognosis. Therefore, we constructed prognostic nomograms based on the results of multivariable COX regression analysis. These nomograms combine the advantages of individual circumstances, tumor characteristics, serum tumor markers, and inflammation-related markers, which can provide individualized prognostic predictions for CRC patients. The results of the C-index and calibration diagram confirmed that these prognostic nomograms had a good prediction accuracy. Randomized internal validation proved that these nomograms have good utility. Compared to the traditional TNM staging system, we found that AALR-based nomograms provided better clinical benefits for both PFS and OS over a 1–5 year period. These nomograms can help to develop individualized risk stratification, follow-up, and treatment strategies for CRC patients.

We demonstrated for the first time that the AALR is a useful tool for predicting long-term outcomes in CRC patients. In addition, AALR could provide a valuable reference for sarcopenia and postoperative complications in CRC patients. We further constructed AALR-based prognostic nomograms, which can be more personalized and convenient to use in clinical practice. However, the current study has several limitations. First, this was a single-center retrospective study, with problems such as a small sample size and patient selection bias. These may have produced statistical deviations. Second, the AALR may be affected by factors such as preoperative laboratory hematological testing techniques and sample collection. Finally, this study lacked an independent validation cohort, which presents an additional limitation. Therefore, prospective, multicenter, large-sample studies should be conducted in the future to further determine the relationship between AALR and CRC patients.

## Conclusion

The AALR is an effective prognostic marker for predicting long-term outcomes and could provide a valuable reference for sarcopenia and postoperative complications in CRC patients. AALR-based nomograms have good predictive accuracy and can be used to identify high-risk patients with poor outcomes.

## Data Availability

The raw data supporting the conclusions of this article will be made available by the authors, without undue reservation.
