# Supplementary material for: Aminotransferase-to-lymphocyte ratio as a valuable prognostic marker for patients with stage I-III colorectal cancer: a retrospective study
Source: Front Oncol. 2024 Nov 18;14:1446557. doi: 10.3389/fonc.2024.1446557 (PMC11609077; doi:10.3389/fonc.2024.1446557)
Supplement: Supplementary file 1 [file DataSheet1.docx]

**Figure S1.** The optimal threshold of AALR in predicting the prognosis of CRC.

**
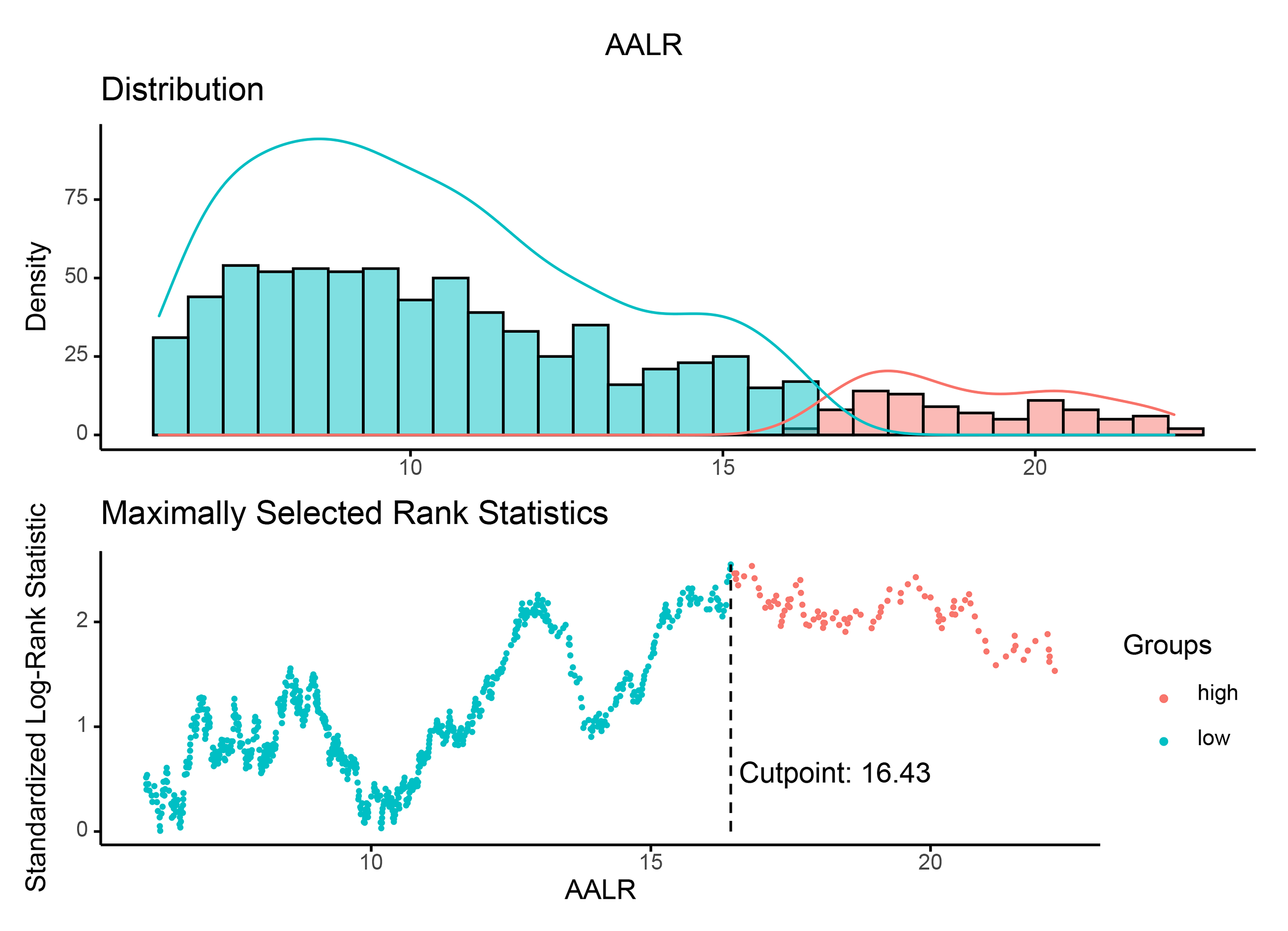
**

**Figure S2.** Restricted cubic spline of AALR in CRC patients.


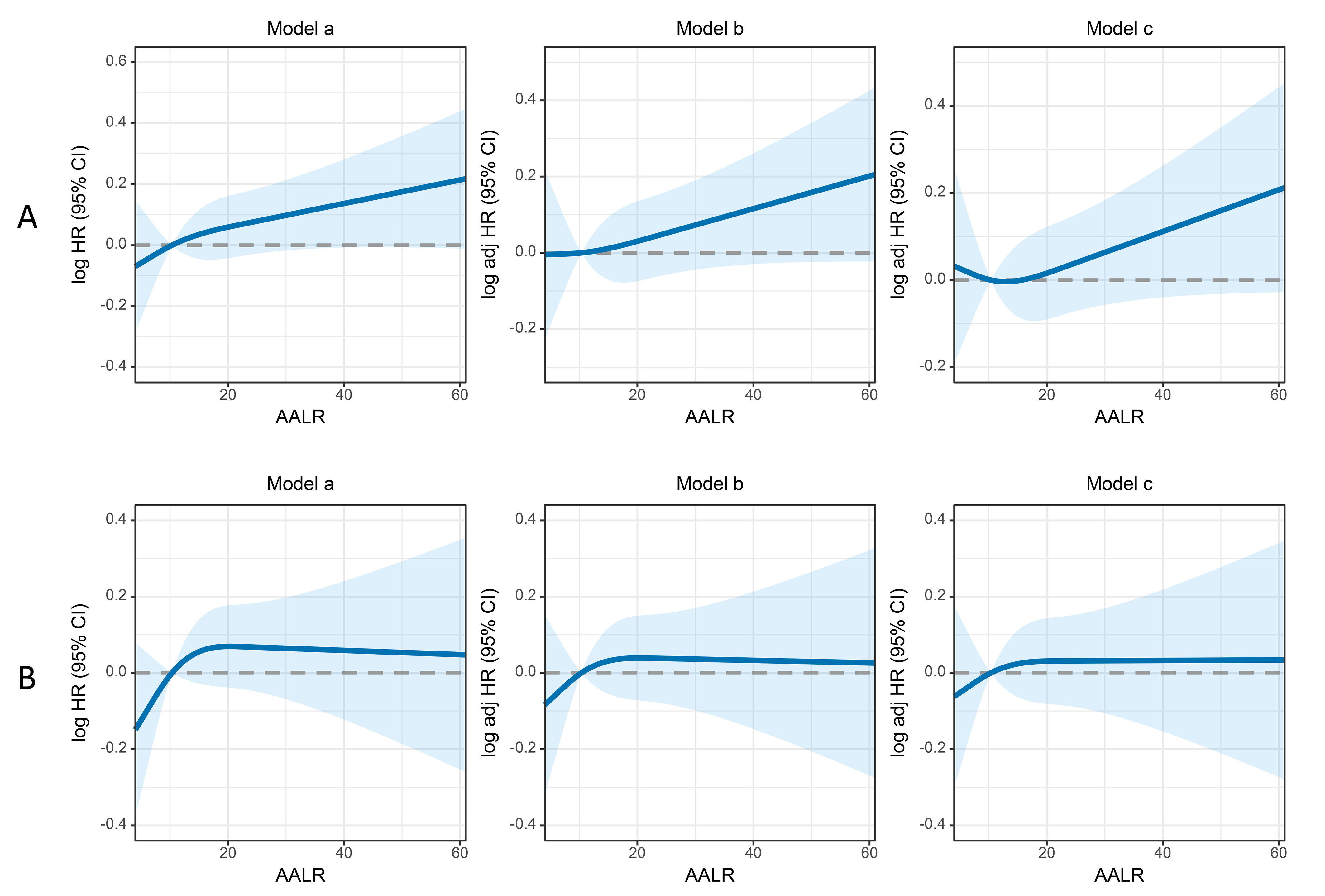


Notes: A, Progression-free survival; B, Overall survival.

Model a: No adjusted.

Model b: Adjusted for gender, age, and BMI.

Model c: Adjusted for gender, age, BMI, hypertension, diabetes, T stage, N stage, tumor location, tumor size, perineural invasion, vascular invasion, macroscopic type, differentiation, radiotherapy, chemotherapy.

**Figure S3.** Stratified survival analysis of AALR based on different TNM stage.


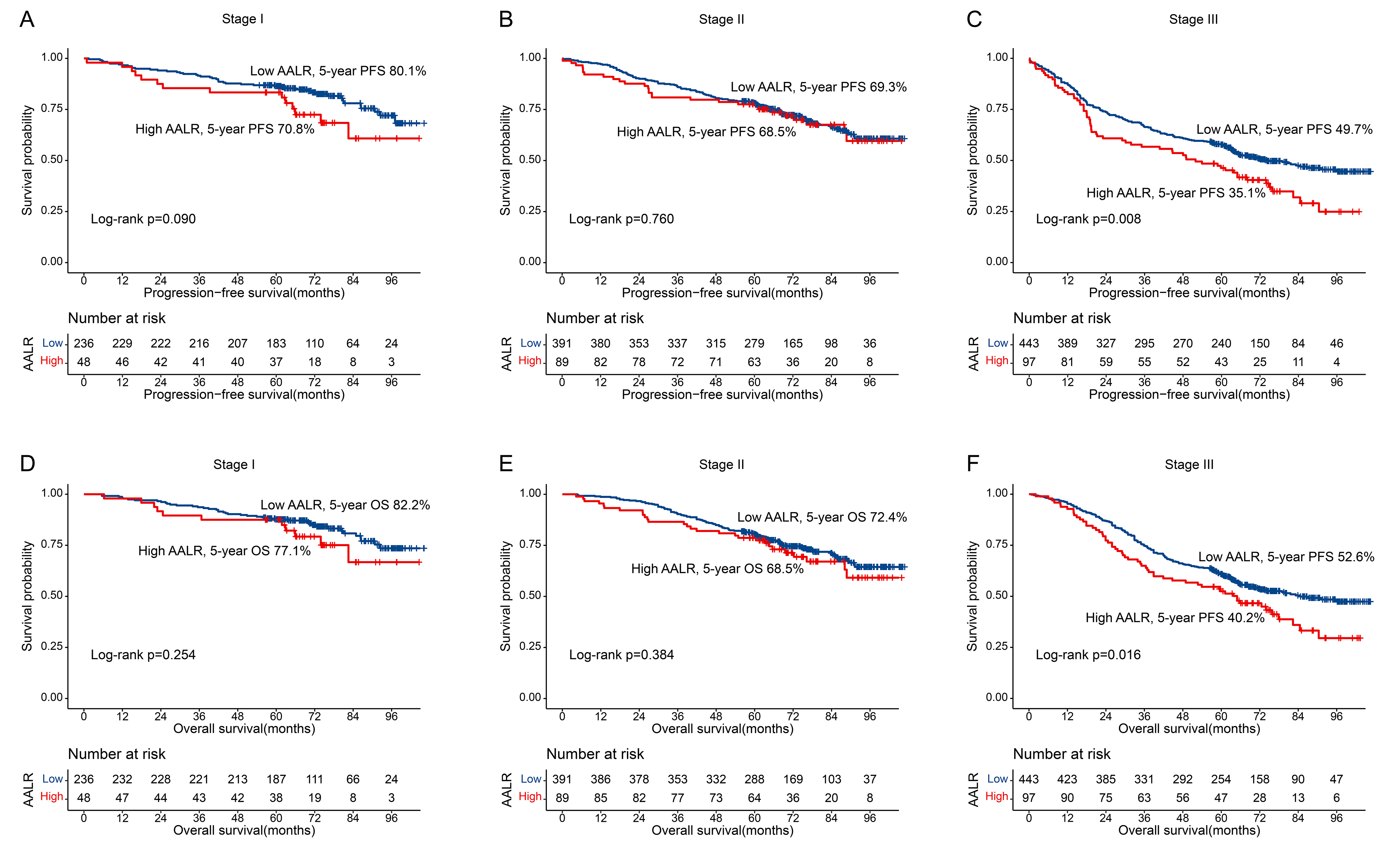


Notes: A, Progression-free survival of stage I; B, Progression-free survival of stage II; C, Progression-free survival of stage III; D, Overall survival of stage I; E, Overall survival of stage II; F, Overall survival of stage III.

**Figure S4.** Calibration curve of the prognostic nomograms.

**
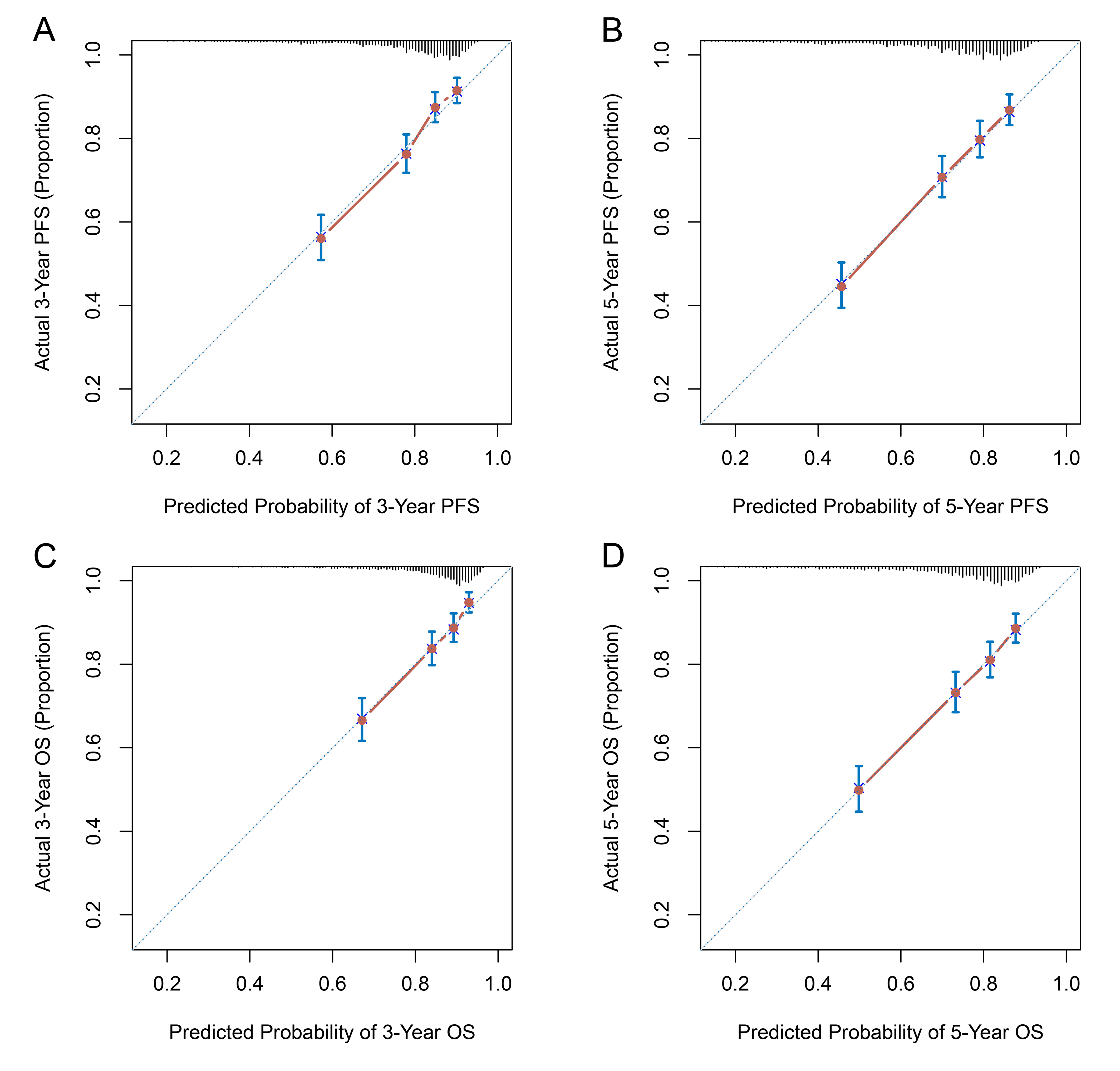
**

Notes: A, 3-year progression-free survival; B, 5-year progression-free survival; C, 3-year overall survival; D, 5-year overall survival.

**Figure S5.** The DCA of PFS/OS nomograms.

**
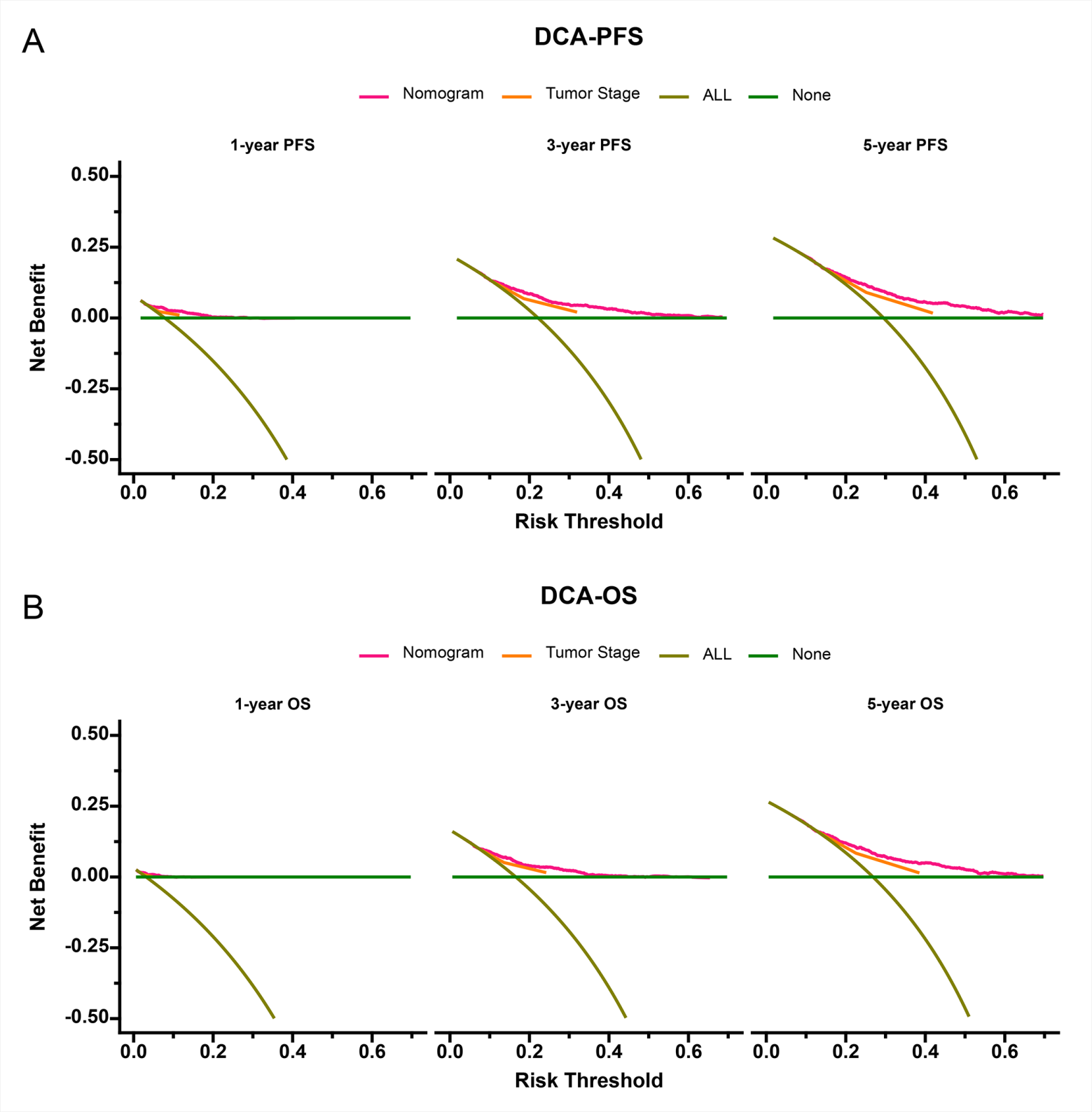
**

**Notes:** A, PFS; B, OS.

**Abbreviation:** PFS, Progression-free survival; OS, Overall survival; DCA, Decision curve analysis.

**Figure S6.** Calibration curve at randomize internal validations.

**
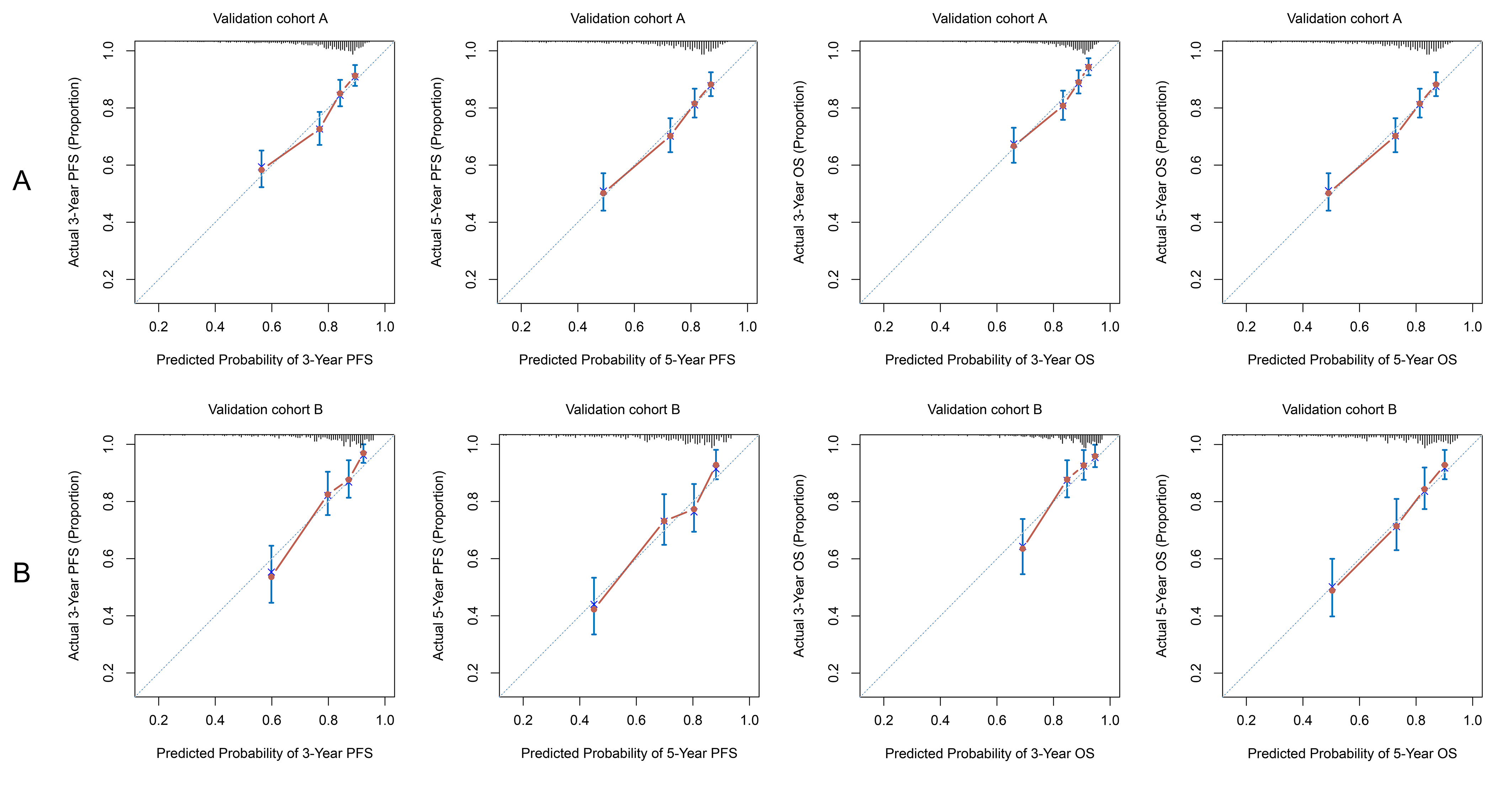
**

**Notes:** A, Validation cohort A; B, Validation cohort B.

**Table S1** Univariate and multivariate Logistic regression analysis of clinicopathological characteristics associated with sarcopenia in CRC patients.

| Clinicopathological characteristics | Univariate analysis | | Multivariate analysis | |
| --- | --- | --- | --- | --- |
|  | HR (95%CI) | P value | HR (95%CI) | P value |
| Sex(Man) | 1.132 (0.850-1.508) | 0.397 |  |  |
| Age (mean (SD)) | 3.385 (2.482-4.617) | <0.001 | 6.724 (4.267 - 10.594) | <0.001 |
| BMI (median [IQR]) |  | <0.001 |  | <0.001 |
| Low BMI |  |  |  |  |
| Normal BMI | 0.052 (0.035-0.076) | <0.001 | 0.032 (0.020-0.052) | <0.001 |
| High BMI | NA | NA | NA | NA |
| Hypertension (Yes) | 1.511 (0.999-2.285) | 0.051 |  |  |
| Diabetes (Yes) | 1.678 (0.853-3.302) | 0.134 |  |  |
| T stage (T3-4) | 1.007 (0.737-1.376) | 0.965 |  |  |
| N stage |  | 0.838 |  |  |
| N0 |  |  |  |  |
| N1 | 1.006 (0.728-1.390) | 0.973 |  |  |
| N2 | 0.885 (0.581-1.349) | 0.570 |  |  |
| Perineural invasion (Yes) | 0.853 (0.517-1.407) | 0.533 |  |  |
| Vascular invasion (Yes) | 0.874 (0.590-1.296) | 0.503 |  |  |
| Macroscopic type |  | 0.035 |  | 0.102 |
| Protrude type |  |  |  |  |
| Infiltrating type | 1.197 (0.716-2.003) | 0.493 | 1.226 (0.615-2.444) | 0.562 |
| Ulcerative type | 0.726 (0.533-0.988) | 0.041 | 0.704 (0.464-1.069) | 0.100 |
| Differentiation (High-medium) | 0.867 (0.563-1.335) | 0.516 |  |  |
| Tumor location (Colon cancer) | 0.868 (0.656-1.148) | 0.320 |  |  |
| Tumor size (≥5cm) | 1.332 (1.007-1.763) | 0.045 | 1.590(1.092-2.314) | 0.016 |
| CEA (High) | 1.204 (0.906-1.601) | 0.201 |  |  |
| AALR (High) | 1.694 (1.213-2.366) | 0.002 | 1.765 (1.120-2.781) | 0.002 |

**Table Note:** CRC, colorectal cancer; BMI, body mass index; AALR, aminotransferase-to-lymphocyte ratio.

**Table S2** Univariate and multivariate Logistic regression analysis of clinicopathological characteristics associated with complication in CRC patients.

| Clinicopathological characteristics | Univariate analysis | | Multivariate analysis | |
| --- | --- | --- | --- | --- |
|  | HR (95%CI) | P value | HR (95%CI) | P value |
| Sex(Man) | 1.002 (0.759-1.324) | 0.988 |  |  |
| Age (mean (SD)) | 1.606 (1.222-2.110) | 0.001 | 1.615 (1.226 - 2.128) | 0.001 |
| BMI (median [IQR]) |  | 0.620 |  |  |
| Low BMI |  |  |  |  |
| Normal BMI | 0.914 (0.607-1.377) | 0.667 |  |  |
| High BMI | 0.812 (0.517-1.274) | 0.364 |  |  |
| Hypertension (Yes) | 1.350 (0.959-1.900) | 0.086 |  |  |
| Diabetes (Yes) | 1.366 (0.816-2.286) | 0.236 |  |  |
| T stage (T3-4) | 1.431 (1.042-1.964) | 0.027 | 1.402 (1.015-1.937) | 0.040 |
| N stage |  | 0.584 |  |  |
| N0 |  |  |  |  |
| N1 | 1.160 (0.851-1.581) | 0.348 |  |  |
| N2 | 1.143 (0.775-1.688) | 0.500 |  |  |
| Perineural invasion (Yes) | 1.502 (0.983-2.296) | 0.060 |  |  |
| Vascular invasion (Yes) | 1.366 (0.965-1.933) | 0.079 |  |  |
| Macroscopic type |  | 0.760 |  |  |
| Protrude type |  |  |  |  |
| Infiltrating type | 1.157 (0.676-1.981) | 0.594 |  |  |
| Ulcerative type | 1.113 (0.819-1.513) | 0.493 |  |  |
| Differentiation (High-medium) | 0.769 (0.526-1.126) | 0.177 |  |  |
| Tumor location (Colon cancer) | 1.270 (0.970-1.663) | 0.082 |  |  |
| Tumor size (≥5cm) | 1.315 (1.004-1.722) | 0.046 | 1.324(1.015-1.937) | 0.040 |
| CEA (High) | 1.215 (0.924-1.598) | 0.164 |  |  |
| AALR (High) | 1.793 (1.300-2.473) | <0.001 | 1.781 (1.286-2.468) | 0.001 |

**Table Note:** CRC, colorectal cancer; BMI, body mass index; AALR, aminotransferase-to-lymphocyte ratio.

**Table S3.** The clinicopathological characteristics of two validation cohorts in CRC patients.

| Clinicopathological characteristics | Validation a  (n = 1009) | Validation b  (n = 432) | P value |
| --- | --- | --- | --- |
| Sex(Man) | 582 (63.5) | 239 (61.6) | 0.548 |
| Age (mean (SD)) | 58.28 (12.95) | 58.38 (13.12) | 0.903 |
| BMI (median (IQR]) | 22.00 (19.94, 24.35) | 22.22 (20.20, 24.80) | 0.121 |
| Hypertension (Yes) | 155 (16.9) | 63 (16.2) | 0.825 |
| Diabetes (Yes) | 58 ( 6.3) | 24 ( 6.2) | 1 |
| T stage (T3-4) | 657 (71.7) | 283 (72.9) | 0.705 |
| N stage |  |  | 0.897 |
| N0 | 537 (58.6) | 227 (58.5) |  |
| N1 | 244 (26.6) | 107 (27.6) |  |
| N2 | 135 (14.7) | 54 (13.9) |  |
| TNM stage (III-IV) |  |  | 0.987 |
| Stage I | 200 (21.8) | 84 (21.6) |  |
| Stage II | 338 (36.9) | 142 (36.6) |  |
| Stage III | 378 (41.3) | 162 (41.8) |  |
| Perineural invasion (Yes) | 88 ( 9.6) | 34 ( 8.8) | 0.708 |
| Vascular invasion (Yes) | 143 (15.6) | 65 (16.8) | 0.666 |
| Macroscopic type |  |  | 0.57 |
| Protrude type | 265 (28.9) | 110 (28.4) |  |
| Infiltrating type | 76 ( 8.3) | 26 ( 6.7) |  |
| Ulcerative type | 575 (62.8) | 252 (64.9) |  |
| Differentiation (Poor) | 801 (87.4) | 335 (86.3) | 0.65 |
| Tumor location (Rectal) | 477 (52.1) | 210 (54.1) | 0.537 |
| Tumor size (median (IQR]) | 4.50 (3.50, 6.00) | 4.50 (3.00, 6.00) | 0.606 |
| CEA (High) | 341 (37.2) | 153 (39.4) | 0.491 |
| Radiotherapy (Yes) | 79 ( 8.6) | 45 (11.6) | 0.116 |
| Chemotherapy (Yes) | 405 (44.2) | 176 (45.4) | 0.749 |
| Death (Yes) | 320 (34.9) | 137 (35.3) | 0.947 |
| HOS (median [IQR]) | 16.50 (11.00, 21.00) | 17.00 (11.00, 21.00) | 0.643 |
| Hospitalization cost (median [IQR]) | 49029.33 (44349.75, 55545.54) | 50142.91 (44980.96, 56758.66) | 0.072 |

**Table Note:** CRC, colorectal cancer; BMI, body mass index; AALR, aminotransferase-to-lymphocyte ratio.
